# Prompt versus deferred conditional cardioversion strategies after early AF recurrence following pulsed field ablation

**DOI:** 10.3389/fcvm.2026.1948373

**Published:** 2026-10-28

**Authors:** Yan Wang

**Affiliations:** Department of Cardiology, Henan Provincial Chest Hospital (Chest Hospital of Zhengzhou University), Zhengzhou, Henan, China

**Keywords:** atrial fibrillation, atrial fibrillation burden, electrical cardioversion, pulsed-field ablation, target trial emulation

## Abstract

**Background:**

The optimal timing of cardioversion for sustained atrial fibrillation (AF) recurrence after pulsed-field ablation (PFA) remains uncertain. We compared prompt and deferred conditional-cardioversion strategies in relation to subsequent AF burden.

**Methods:**

This retrospective cohort study included 214 adults with continuously monitored AF lasting at least 24 h during the 8-week blanking period after first-time PFA. At eligibility, each patient was cloned to two dynamic strategies: attempting cardioversion within 48 h if AF persisted or withholding nonrescue cardioversion for 7 days. Strategy deviations were artificially censored and inverse-probability weighted. The primary per-protocol estimand was the strategy difference in cumulative AF days from postablation days 57 through 365.

**Results:**

Artificial censoring occurred in 35.5% and 59.3% of prompt- and deferred-strategy clones, respectively; the corresponding effective sample sizes were 124.7 and 72.9. Standardized mean cumulative AF exposure was 17.9 days under the prompt strategy and 30.5 days under the deferred strategy (mean difference, −12.6 days; 95% CI, −21.6 to −4.6; mean ratio, 0.59; 95% CI, 0.40–0.84). The risk of first sustained recurrence after day 56 was 30.7% vs. 44.1% (weighted hazard ratio, 0.64; 95% CI, 0.45–0.91). Repeat cardioversion and AF-related hospitalization were also less frequent under the prompt strategy. Continuation of the qualifying episode accounted for approximately 39% of the primary difference; after excluding this component, AF burden from distinct subsequent episodes remained lower under the prompt strategy (mean difference, −7.7 days; 95% CI, −14.9 to −1.1). Safety events were uncommon.

**Conclusions:**

A strategy of attempting prompt cardioversion when AF persisted was associated with lower subsequent AF burden than temporary deferral. Randomized validation is required before defining an optimal cardioversion window or making firm management recommendations.

## Introduction

Catheter ablation is an established rhythm-control treatment for symptomatic atrial fibrillation (AF), with pulmonary-vein isolation remaining the procedural cornerstone. Pulsed-field ablation (PFA) has rapidly expanded this field by producing nonthermal myocardial injury through irreversible electroporation. Prospective multicenter studies have demonstrated clinically meaningful 1-year effectiveness with a favorable procedural safety profile across paroxysmal and persistent AF populations (1, 2). In a randomized comparison, PFA was noninferior to conventional thermal ablation for paroxysmal AF (3), while the EU-PORIA registry supported its feasibility and effectiveness in routine practice (4).

Despite these advances, early atrial tachyarrhythmia recurrence remains common after PFA. Recent randomized comparisons with cryoballoon ablation and sham control have strengthened the evidence for PFA but have not resolved how sustained recurrence should be managed during early healing (5, 6). Such recurrence may reflect transient edema, inflammation, autonomic perturbation, or temporary conduction recovery and is therefore not automatically classified as treatment failure. Clinicians must consequently balance two plausible approaches when sustained AF occurs: early restoration of sinus rhythm to limit ongoing arrhythmia exposure, or short-term deferral to allow spontaneous conversion and avoid an unnecessary procedure.

Evidence guiding that decision is limited. Most PFA studies have evaluated the ablation technology, procedural safety, or freedom from recurrent arrhythmia rather than the timing of cardioversion after a sustained early recurrence. Conventional observational comparisons are also vulnerable to confounding by indication because symptom severity, ventricular rate, heart-failure status, anticoagulation, and evolving rhythm-control treatment may influence both cardioversion timing and subsequent outcomes. Intermittent rhythm assessment further obscures the total burden of recurrent AF. A clinically useful comparison therefore requires explicit dynamic strategies, a common time zero, adjustment for time-varying treatment selection, and continuous rhythm monitoring.

Therefore, we compared a strategy of attempting cardioversion within 48 h if AF persisted with a strategy of withholding nonrescue cardioversion for 7 days. The primary objective was to estimate the per-protocol strategy difference in cumulative AF days from postablation days 57 through 365.

## Methods

### Study design and data sources

This retrospective cohort study was conducted at Henan Chest Hospital from January 2023 through May 2025. The institutional review board approved the study; all patients provided written informed consent, and the study conformed to the Declaration of Helsinki.

### Study population

Adults were eligible if they underwent first-time PFA for paroxysmal or persistent AF, achieved sinus rhythm, subsequently developed continuously documented AF lasting at least 24 h during the prespecified 8-week blanking period, and had monitoring capable of quantifying both AF and analyzable time. Time zero was when AF reached 24 h. Consistent with contemporary consensus recommendations, the 8-week boundary separated early management from outcome ascertainment; time zero was restricted to postablation days 3–49 so that both strategy windows ended by day 56. Monitoring was provided by implantable cardiac monitors, pacemakers, defibrillators or cardiac resynchronization devices, or validated continuously worn ECG systems.

Exclusion criteria were previous left atrial ablation; failure to restore sinus rhythm after PFA; isolated atrial flutter or atrial tachycardia; emergency cardioversion at time zero; left atrial thrombus or inadequate anticoagulation; an acute reversible AF trigger; repeat ablation before eligibility; or imprecise rhythm or cardioversion timing.

### Index PFA procedure

Index PFA was performed under general anesthesia or deep sedation with uninterrupted oral anticoagulation. Intravenous heparin targeted an activated clotting time of 300–350 s. Pulmonary-vein isolation (PVI) was required in all patients.

FARAPULSE procedures used a 31- or 35 mm FARAWAVE catheter with eight 2.5 s, 2.0 kV applications per vein (four basket and four flower, with rotation). PulseSelect procedures used a 25 mm loop catheter with four ostial and four antral applications per vein, repositioned between applications. Additional applications treated residual conduction. PVI was confirmed by entrance block and, when feasible, exit block after at least 20 min.

Posterior-wall ablation was considered only for persistent AF with AF duration at least 6 months, left atrial volume index at least 40 mL/m^2^, or posterior-wall low-voltage substrate. Overlapping applications were delivered with the FARAWAVE flower configuration or under electroanatomic guidance with PulseSelect; the endpoint was posterior-wall entrance block. Other lesion sets were limited to documented or inducible typical flutter, atrial tachycardia, mitral-isthmus, or superior vena cava substrates.

### Treatment strategies

The target trial compared two dynamic strategies initiated at time zero. The prompt strategy attempted synchronized cardioversion within 48 h only if AF persisted; the deferred strategy withheld nonrescue cardioversion for 7 days and permitted it thereafter if AF persisted. Rescue cardioversion was permitted at any time. Spontaneous conversion remained strategy-adherent, whereas adherence among patients with persistent AF depended on the timing of the attempt, irrespective of success.

### Rescue cardioversion and procedural management

During the 7-day deferred window, rescue cardioversion was urgent cardioversion for hemodynamic instability; worsening heart failure requiring intravenous therapy; uncontrolled ventricular rate despite treatment; refractory severe symptoms, syncope, or myocardial ischemia; or an AF-attributable unplanned hospitalization requiring rhythm restoration. Planned cardioversion for otherwise stable AF was nonrescue. Rescue indications developing after time zero were allowed and did not trigger artificial censoring.

Cardioversion used synchronized biphasic shocks under monitored sedation. Immediate restoration was sinus rhythm lasting at least 30 s; early reinitiation was AF recurrence within 10 min; and acute success was sinus rhythm after the 30 min observation period. Failure or early reinitiation did not alter strategy adherence.

Therapeutic anticoagulation was continued after PFA. TEE within 24 h before cardioversion was required when uninterrupted treatment during the preceding 3 weeks could not be verified or clinical concern for left atrial appendage thrombus existed; cardioversion was postponed if thrombus was detected. Anticoagulation was uninterrupted for cardioversion and continued for at least 4 weeks afterward.

### Outcomes

The primary outcome was time-weighted AF burden from postablation days 57–365, calculated as AF minutes divided by analyzable monitoring minutes. Native device algorithms screened for irregular R-R intervals without consistent *P* waves, and wearable ECG classifications were harmonized to the same study definition with a common minimum episode duration of 2 min. Eligibility required at least 80% analyzable coverage; signal loss, device interruption, and nonwear were excluded from the denominator, without imputing AF during gaps. Observation weights accounted for partially observed intervals. All eligibility-defining and uncertain episodes and a random 20% sample of other detections were independently reviewed by electrophysiologists, with disagreements resolved by consensus.

Preablation burden was measured over the 90 days before PFA using the same episode threshold, coverage requirement, gap handling, and adjudication. AF burden was converted to cumulative AF days by multiplying the burden proportion by the outcome-window duration. If the qualifying episode continued beyond day 56, that time contributed to the primary outcome.

The causal estimand was the per-protocol total effect of implementing the prompt conditional-cardioversion strategy vs. the deferred strategy on mean cumulative AF days. It therefore included downstream management after the strategy window and did not isolate the biological effect of receiving or successfully undergoing cardioversion.

Antiarrhythmic drug class, dose, and start and stop dates were recorded at time zero, during each 24 h strategy interval, and through day 365. Modifications were initiation, discontinuation, agent or class switching, or a dose change of at least 25%. Temporary pericardioversion doses were recorded as interval-specific therapy but counted as chronic modifications only if the maintenance prescription changed. Therapy during strategy assignment entered the censoring models; later drug changes, cardioversion, and repeat ablation were treated as downstream mediators and not adjusted for in the primary analysis.

Secondary outcomes were at least 90% reduction from preablation AF burden, first sustained recurrence after day 56, repeat cardioversion, repeat ablation, AF-related hospitalization, AF-burden thresholds, total atrial tachyarrhythmia burden, and weekly AF burden during days 8–21 after time zero. AF-related hospitalization required an unplanned admission longer than 24 h or crossing a calendar day, with documented AF as the principal cause or precipitant requiring acute treatment. Heart-failure hospitalization additionally required objective congestion and intensified treatment. Ischemic stroke required a focal deficit lasting at least 24 h or imaging-confirmed infarction; systemic embolism required acute extracranial arterial occlusion; and major bleeding was fatal or critical-site bleeding, a hemoglobin decrease of at least 2 g/dL, or transfusion of at least 2 units. Hospitalizations were assessed from day 8 after time zero; stroke or systemic embolism, major bleeding, and death were assessed from time zero, all through postablation day 365.

### Covariates

Baseline covariates comprised demographics, AF type and duration, preablation burden, previous cardioversion, comorbidities, cardiac structure and function, and rhythm- and rate-control medications. Procedural covariates were PFA platform, posterior-wall ablation, intraprocedural cardioversion, operator, and year. Time-zero and updated covariates included recurrence timing, AF duration and recent burden, ventricular rate, symptoms, acute-care and heart-failure status, potassium, magnesium, creatinine, infection or inflammation, anticoagulation, and rhythm- and rate-control therapy.

### Missing data

Missing covariates were multiply imputed before cloning using chained equations (20 datasets). The imputation model included all censoring and monitoring-model variables, cardioversion timing, censoring indicators, and the primary outcome; time-varying values were imputed in person-interval format using interval and lagged information. The complete analysis was repeated in each dataset, with imputation repeated within each patient-level bootstrap sample.

### Target trial emulation and statistical analysis

Each patient was cloned at time zero and assigned to both strategies. Clones were artificially censored when observed care became incompatible: prompt-strategy clones if persistent AF was not cardioverted within 48 h, and deferred-strategy clones if nonrescue cardioversion occurred within 7 days. Spontaneous conversion did not trigger censoring.

Stabilized inverse-probability-of-censoring weights were estimated in 24 h intervals using separate strategy-specific pooled logistic models. Denominator models included baseline and lagged time-varying covariates; numerator models included baseline covariates and interval indicators. Continuous variables used restricted cubic splines with three knots, categorical variables used indicator terms, and time used interval indicators. Treatment-adherence and observation weights were multiplied and truncated at the 1st and 99th percentiles. Positivity was assessed from compatibility probabilities, weight distributions, and effective sample size; balance was assessed using absolute standardized mean differences. Cloning, weight estimation, truncation, and outcome modeling were repeated in 1,000 patient-level bootstrap samples.

A weighted two-part model estimated the probability of any AF and burden among patients with nonzero burden. We report standardized means, mean differences, and mean ratios with bootstrap 95% CIs. Binary outcomes used standardized risk differences and risk ratios; time-to-event outcomes used weighted survival models.

Exploratory daily landmark trials compared cardioversion within the next 24 h with continued deferral among patients still in AF without previous cardioversion; pooled estimates described the association by time since eligibility. A separate prespecified analysis tested effect modification by postablation recurrence timing using restricted cubic splines and strategy-by-timing interactions.

### Sensitivity analyses

Sensitivity analyses varied the sustained-AF threshold, prompt window, weight truncation, common-support restriction, and blanking period; restricted monitoring coverage, stable antiarrhythmic therapy, and verified anticoagulation; and excluded acute concurrent conditions. We partitioned burden into continuation of the qualifying episode and distinct subsequent episodes, excluded qualifying-episode AF, and delayed outcome ascertainment to days 90 and 120. A supportive analysis censored and reweighted follow-up at the first post-strategy drug modification, cardioversion, or repeat ablation. Complete-case and quantitative bias analyses were also performed.

Analyses used R version 4.5.2 (R Foundation for Statistical Computing, Vienna, Austria). Tests were two-sided, with *P* < 0.05 denoting statistical significance.

## Results

### Baseline characteristics

Of 2,140 screened patients, 214 were included and cloned to both strategies (Supplementary Figure S1). Within 48 h, 109 underwent cardioversion and 29 converted spontaneously. Among the remaining 76, 18 underwent nonrescue cardioversion by day 7, 24 converted spontaneously, and 34 remained in AF. Thus, 138 records remained compatible with the prompt strategy and 87 with the deferred strategy; the 29 early spontaneous conversions were compatible with both.

Mean age was 64.3 years, 42.5% were women, and 46.3% had persistent AF. Median preablation AF burden was 28.6% (IQR, 14.2%–51.8%). Active antiarrhythmic therapy was present in 69.6% at index PFA and 75.7% at time zero. The qualifying recurrence was symptomatic in 72.9%, including EHRA class III or IV symptoms in 51.9%. Implantable monitoring was used in 71.0%; all patients had at least 80% coverage, and 86.9% had at least 90% coverage during days 57–365. The predominant pre- and postablation monitoring modality was unchanged in 97.2%. Overall, 186 patients (86.9%) had complete covariate data; individual-variable missingness ranged from 0% to 6.5% (Supplementary Table S1).

FARAPULSE was used in 132 patients (61.7%) and PulseSelect in 82 (38.3%). All pulmonary veins were acutely isolated; 30 patients (14.0%) required additional applications. Posterior-wall ablation was performed in 43 patients (20.1%), all with persistent AF, and other adjunctive lesion sets were uncommon (Supplementary Table S2).

Cardioversion for the qualifying recurrence was attempted in 154 patients: 109 within 48 h, 18 between 48 h and day 7, and 27 after day 7. Of the 34 patients still in AF at day 7, 27 were later cardioverted, 5 converted spontaneously, and 2 continued rate-control management. No rescue indication occurred during days 0–7. Immediate sinus rhythm was restored in 149 of 154 attempts (96.8%); early reinitiation occurred in 8 of 149 (5.4%), and acute success was 94.2%. TEE was used before 14 procedures, detected no thrombus, and all cardioverted patients continued uninterrupted anticoagulation for at least 4 weeks (Supplementary Table S3).

### Target trial emulation and weighting diagnostics

Artificial censoring affected 76 prompt-strategy clones (35.5%) and 127 deferred-strategy clones (59.3%). The median compatibility probabilities were 0.65 and 0.42, respectively. After weight truncation, effective sample sizes were 124.7 and 72.9 (58.3% and 34.1% of assigned clones), indicating weaker empirical support for the deferred strategy. All postweighting absolute standardized mean differences were below 0.10 (maximum, 0.057) (Supplementary Table S4).

During follow-up, 46 patients (21.5%) had at least one antiarrhythmic drug modification; the standardized risks were 19.3% under the prompt strategy and 26.7% under the deferred strategy (risk difference, −7.4 percentage points; 95% CI, −16.0 to 1.4). Subsequent cardioversion occurred in 22 of 138 prompt-compatible and 22 of 87 deferred-compatible clones; repeat ablation occurred in 12 and 11, respectively (Supplementary Table S5).

### Primary outcomes

Standardized mean cumulative AF exposure was 17.9 days (95% CI, 12.6–24.0) under the prompt strategy and 30.5 days (95% CI, 23.1–39.1) under the deferred strategy, giving the primary mean difference of −12.6 days (95% CI, −21.6 to −4.6). Corresponding mean burdens were 5.8% and 9.9% (difference, −4.1 percentage points; 95% CI, −7.0 to −1.5), with a mean ratio of 0.59 (95% CI, 0.40–0.84) (Table 1).

**Table 1:** Weighted analysis of cumulative atrial fibrillation burden.

Outcome measure	Prompt strategy	Deferred strategy	Adjusted absolute effect^a^	Adjusted relative effect^b^	*P* value
Two-part model: occurrence component					
Any AF during days 57–365, %	42.4 (35.1–49.8)	57.6 (49.6–65.2)	−15.2 (−25.6 to −4.7) percentage points	RR, 0.74 (0.61–0.90)	0.006
Two-part model: positive-burden component					
Mean AF burden among patients with nonzero burden, %	13.7 (10.6–17.5)	17.2 (13.7–21.3)	−3.5 (−7.0 to −0.4) percentage points	MR, 0.80 (0.62–0.98)	0.029
Marginal primary-outcome estimates					
Adjusted mean time-weighted AF burden, %	5.8 (4.1–7.8)	9.9 (7.5–12.7)	−4.1 (−7.0 to −1.5) percentage points	MR, 0.59 (0.40–0.84)	0.004
Cumulative AF days during the 308-day outcome period^c^	**17.9** **(12.6–24.0)**	**30.5** (**23.1–39.1)**	**−12.6 (−21.6 to −4.6) days**	**MR, 0.59 (0.40–0.84)**	**0**.**004**

Data are adjusted estimates with 95% CIs derived from 1,000 patient-level nonparametric bootstrap samples.

a Prompt minus deferred strategy; the cumulative AF-day difference was the primary estimand.

b Relative effects are risk ratios (RRs) for binary outcomes and mean ratios (MRs) for continuous outcomes.

c Calculated as the adjusted mean AF-burden proportion multiplied by 308 days.

Weight models included age, sex, BMI, AF type and duration, preablation AF burden, previous cardioversion, comorbidities, cardiac structure and function, medications, PFA system, posterior-wall ablation, intraprocedural cardioversion, operator, calendar year, recurrence timing, recent AF burden, ventricular rate, symptom severity, acute-care status, heart-failure status, laboratory measurements, infection or inflammation, and anticoagulation status.

Bold values indicates *p* < 0.05.

### Secondary outcomes

At least 90% reduction from preablation burden occurred in 69.1% under the prompt strategy and 54.7% under the deferred strategy (risk difference, 14.4 percentage points; 95% CI, 3.9–24.6). Results were unchanged after excluding six patients who changed monitoring systems. First sustained recurrence after day 56 was less frequent under the prompt strategy (30.7% vs. 44.1%; HR, 0.64; 95% CI, 0.45–0.91), as were repeat cardioversion (15.8% vs. 24.9%; HR, 0.60; 95% CI, 0.37–0.96) and AF-related hospitalization (6.9% vs. 13.8%; HR, 0.47; 95% CI, 0.23–0.95). The repeat-ablation estimate was imprecise (8.7% vs. 13.1%; HR, 0.64; 95% CI, 0.34–1.19) (Table 2).

**Table 2:** Weighted secondary, safety, and supplementary outcomes.

Outcome	Prompt strategy, % (95% CI)	Deferred strategy, % (95% CI)	Risk difference, percentage points (95% CI)	Risk ratio (95% CI)	Weighted HR (95% CI)
Secondary outcomes					
≥90% reduction in AF burden	69.1 (62.1–75.6)	54.7 (46.4–62.8)	14.4 (3.9–24.6)	1.26 (1.06–1.52)	—
First sustained recurrence after day 56^a^	30.7 (24.0–38.0)	44.1 (36.0–52.5)	−13.4 (−24.1 to −2.9)	0.70 (0.53–0.90)	0.64 (0.45–0.91)
Repeat cardioversion^b^	15.8 (10.8–21.8)	24.9 (18.1–32.5)	−9.1 (−18.1 to −0.6)	0.63 (0.42–0.94)	0.60 (0.37–0.96)
Repeat ablation^b^	8.7 (5.0–13.6)	13.1 (8.2–19.1)	−4.4 (−11.3 to 2.0)	0.66 (0.38–1.12)	0.64 (0.34–1.19)
AF-related hospitalization^b^	6.9 (3.5–11.2)	13.8 (8.8–19.8)	−6.9 (−13.9 to −0.8)	0.50 (0.27–0.91)	0.47 (0.23–0.95)
Safety outcomes					
Heart-failure hospitalization^b^	3.3 (1.3–6.3)	5.8 (2.8–9.8)	−2.5 (−7.2 to 1.6)	0.57 (0.23–1.30)	0.55 (0.20–1.51)
Ischemic stroke or systemic embolism^c^	1.7 (0.5–3.7)	1.4 (0.3–3.4)	0.3 (−2.0 to 2.6)	1.21 (0.34–4.03)	1.23 (0.27–5.55)
Major bleeding^c^	2.5 (0.9–4.9)	2.2 (0.7–4.7)	0.3 (−2.8 to 3.3)	1.14 (0.39–3.33)	1.16 (0.34–3.95)
All-cause death^c^	1.2 (0.2–3.0)	1.5 (0.3–3.6)	−0.3 (−2.7 to 1.7)	0.80 (0.18–3.18)	0.78 (0.15–4.13)
AF-burden thresholds					
AF burden ≥0.1%	39.8 (32.8–47.2)	55.0 (47.0–62.8)	−15.2 (−25.6 to −4.8)	0.72 (0.59–0.88)	—
AF burden ≥1%	31.9 (25.3–39.1)	45.4 (37.5–53.5)	−13.5 (−23.8 to −3.5)	0.70 (0.55–0.89)	—
AF burden ≥5%	21.6 (15.9–28.1)	33.2 (26.0–40.9)	−11.6 (−20.9 to −2.6)	0.65 (0.47–0.88)	—
Continuous supplementary outcome	Prompt strategy (95% CI)	Deferred strategy (95% CI)	Mean difference (95% CI)	Mean ratio (95% CI)	
Total AF/AFL/AT burden, %	6.3 (4.5–8.4)	10.6 (8.1–13.5)	−4.3 (−7.4 to −1.6)	0.59 (0.40–0.84)	
AF burden during days 8–14, %	8.1 (5.8–10.8)	15.7 (12.0–19.8)	−7.6 (−12.2 to −3.1)	0.52 (0.34–0.76)	
AF burden during days 15–21, %	5.9 (4.0–8.2)	11.9 (8.7–15.7)	−6.0 (−10.0 to −2.3)	0.50 (0.31–0.76)	

Values are standardized weighted estimates with 95% CIs derived from 1,000 patient-level bootstrap samples.

a Risk period: postablation days 57–365.

b Risk period: day 8 after time zero through postablation day 365.

c Risk period: time zero through postablation day 365.

Hospitalization meant an unplanned admission lasting more than 24 h or crossing a calendar day. AF-related hospitalization required documented AF as the principal cause or precipitant requiring acute therapy; heart-failure hospitalization required compatible symptoms or signs, objective congestion, and intensified therapy. Ischemic stroke required a focal neurologic deficit lasting at least 24 h or imaging-confirmed infarction; systemic embolism required confirmed acute extracranial arterial occlusion. Major bleeding met International Society on Thrombosis and Hemostasis criteria: fatal or symptomatic critical-site bleeding, a hemoglobin decrease of at least 2 g/dL, or transfusion of at least 2 units. All-cause death was death from any cause. Only the first event per endpoint was analyzed; AF-related and heart-failure hospitalizations could overlap. HR indicates hazard ratio; AF, atrial fibrillation; AFL, atrial flutter; and AT, atrial tachycardia.

AF-burden thresholds and total atrial tachyarrhythmia burden consistently favored the prompt strategy. Weekly AF burden was 8.1% vs. 15.7% during days 8–14 and 5.9% vs. 11.9% during days 15–21; the strategy-by-week interaction was not significant (*P* = 0.58) (Figure 1 and Table 2).

**Figure 1:**
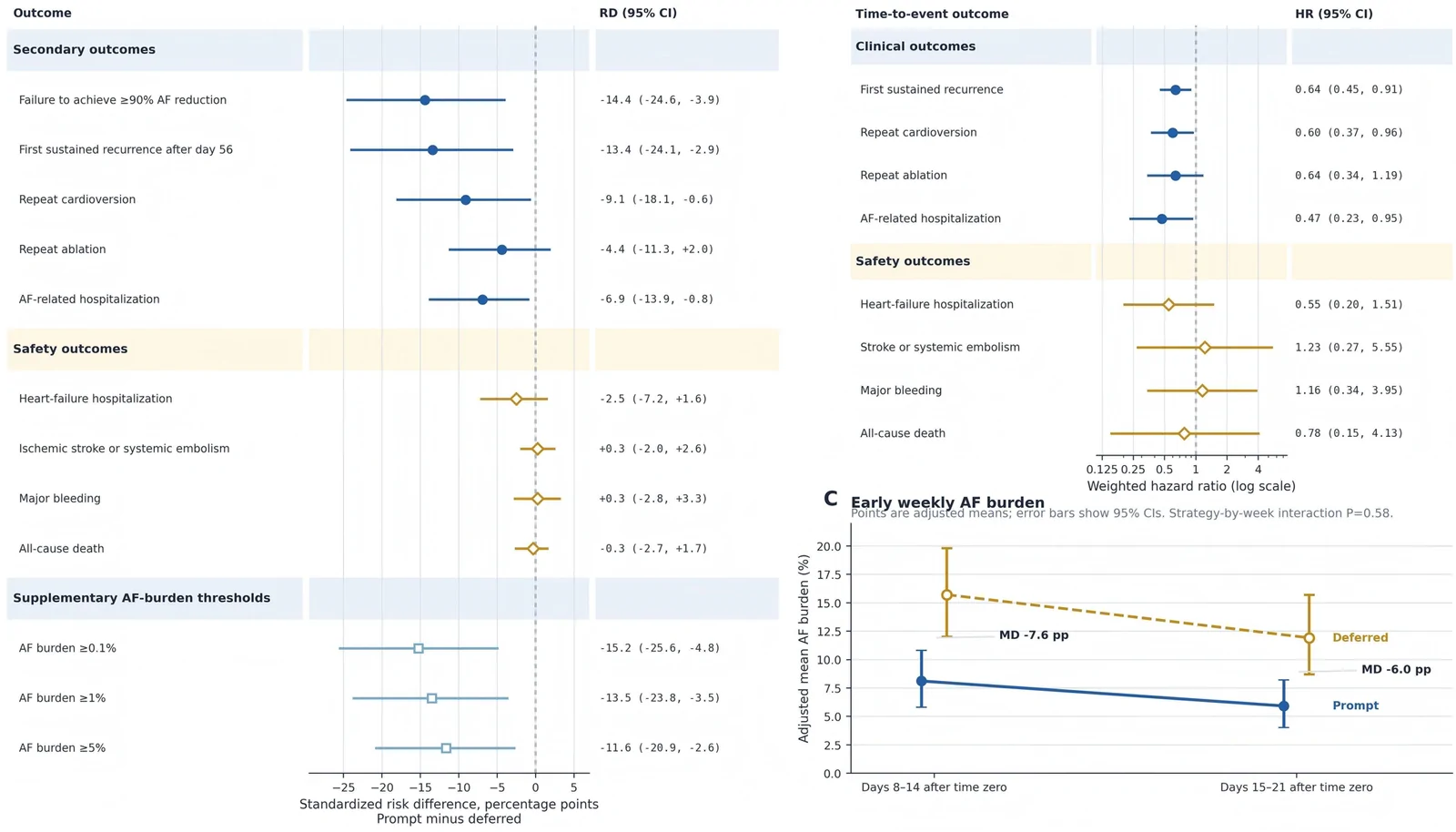
Secondary, safety, and early rhythm-burden outcomes under prompt versus deferred strategies.

Safety events were uncommon. The prompt and deferred estimates were 3.3% vs. 5.8% for heart-failure hospitalization, 1.7% vs. 1.4% for ischemic stroke or systemic embolism, 2.5% vs. 2.2% for major bleeding, and 1.2% vs. 1.5% for death; all 95% CIs included the null (Table 2).

### Exploratory landmark trials and effect modification

In the exploratory landmark analysis, the estimated prompt-strategy difference was −15.0 AF days (95% CI, −25.3 to −6.3) at eligibility and attenuated to −0.8 days (95% CI, −15.4 to 12.1) by day 6. The descriptive average attenuation was 2.3 AF days per later 24 h landmark (95% CI, 0.7–3.9; *P* = 0.005). The strategy effect did not significantly vary by postablation recurrence timing (overall interaction *P* = 0.48; nonlinear interaction *P* = 0.77) (Figure 2).

**Figure 2:**
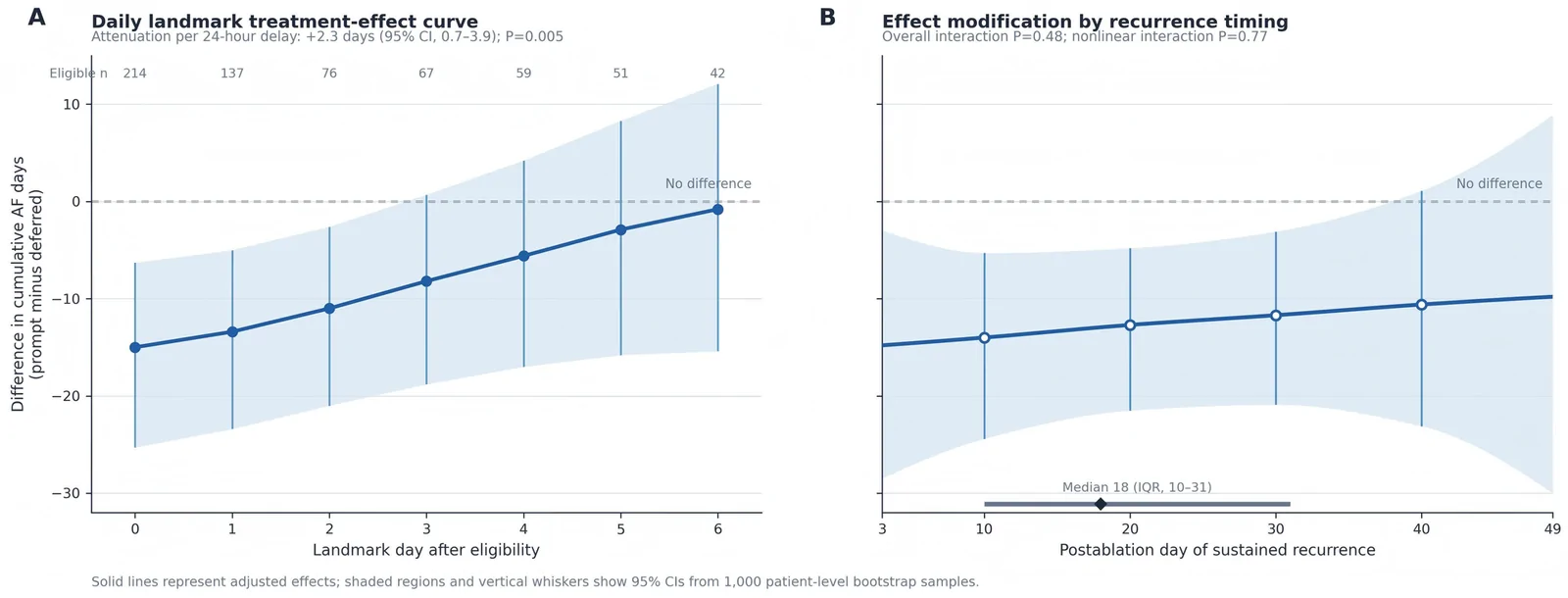
Landmark-trial estimates and effect modification by recurrence timing. **(A)** presents pooled estimates from the sequential daily landmark strategies comparing an attempt at cardioversion within the next 24 h if AF persisted with continued deferral. **(B)** shows the adjusted treatment-effect curve according to the postablation timing of sustained recurrence. Solid lines represent adjusted differences in cumulative AF days; shaded areas and vertical whiskers indicate 95% CIs derived from 1,000 patient-level bootstrap samples. Negative estimates favor prompt strategy. The horizontal bar and diamond in **(B)** indicate the IQR and median recurrence timing, respectively.

### Sensitivity and bias analyses

Results were consistent across alternative recurrence and prompt-window definitions, monitoring, therapy and anticoagulation restrictions, exclusions of acute conditions, weighting and common-support specifications, and 6-week or 90-day blanking periods (mean differences, −9.3 to −14.8 days). Complete-case analysis (*n* = 186) yielded a difference of −12.3 days (95% CI, −21.7 to −4.1) (Table 3).

**Table 3:** Sensitivity analyses of cumulative AF burden.

Analysis	Patients, n	Prompt, adjusted AF days	Deferred, adjusted AF days	Mean difference, days (95% CI)	Mean ratio (95% CI)
Primary analysis	214	17.9	30.5	−12.6 (−21.6 to −4.6)	0.59 (0.40–0.84)
Qualifying-episode decomposition					
Continuation of qualifying episode	214	0.9	5.8	−4.9 (−7.8 to −2.3)	0.16 (0.05–0.40)
Distinct subsequent AF episodes only	214	17.0	24.7	−7.7 (−14.9 to −1.1)	0.69 (0.47–0.97)
Delayed outcome windows					
Postablation days 90–365	214	15.1	23.3	−8.2 (−15.4 to −1.7)	0.65 (0.44–0.94)
Postablation days 120–365	214	12.7	19.0	−6.3 (−12.2 to −0.8)	0.67 (0.44–0.97)
Alternative sustained-AF definition					
AF lasting ≥12 h	247	18.6	30.2	−11.6 (−20.4 to −3.8)	0.62 (0.43–0.88)
AF lasting ≥48 h	181	16.7	30.8	−14.1 (−24.0 to −5.0)	0.54 (0.35–0.80)
Alternative prompt-cardioversion window					
Within 24 h	214	16.9	31.7	−14.8 (−25.1 to −5.3)	0.53 (0.34–0.79)
Within 72 h	214	19.0	30.1	−11.1 (−19.8 to −3.1)	0.63 (0.44–0.89)
Restricted cohorts					
Implantable monitoring only	152	15.8	28.5	−12.7 (−22.6 to −3.8)	0.55 (0.35–0.83)
Monitoring coverage ≥90%	186	17.2	29.7	−12.5 (−21.8 to −4.2)	0.58 (0.39–0.84)
Stable antiarrhythmic therapy	168	16.9	29.4	−12.5 (−22.2 to −3.6)	0.57 (0.37–0.84)
Verified therapeutic anticoagulation	201	17.7	30.0	−12.3 (−21.4 to −4.2)	0.59 (0.40–0.85)
Exclusion of concurrent conditions					
Excluding infection or inflammation	201	17.5	30.0	−12.5 (−21.7 to −4.3)	0.58 (0.39–0.84)
Excluding electrolyte disturbance	198	17.4	29.8	−12.4 (−21.6 to −4.1)	0.58 (0.39–0.85)
Excluding acute heart-failure exacerbation	192	17.1	29.3	−12.2 (−21.6 to −3.8)	0.58 (0.38–0.85)
Excluding any of the above conditions	171	16.4	28.7	−12.3 (−22.1 to −3.4)	0.57 (0.36–0.84)
Alternative weighting specifications					
No weight truncation	214	17.4	30.6	−13.2 (−23.2 to −4.1)	0.57 (0.37–0.83)
Truncation at 2.5th/97.5th percentiles	214	17.8	30.3	−12.5 (−21.4 to −4.6)	0.59 (0.40–0.84)
Truncation at 5th/95th percentiles	214	18.2	29.8	−11.6 (−19.7 to −4.1)	0.61 (0.43–0.86)
Common-support restriction	207	17.7	29.9	−12.2 (−20.9 to −4.2)	0.59 (0.40–0.84)
Alternative blanking periods					
6-week blanking period	174	18.4	32.1	−13.7 (−23.9 to −4.7)	0.57 (0.37–0.84)
90-day blanking period	271	15.6	24.9	−9.3 (−16.8 to −1.9)	0.63 (0.44–0.90)
Complete-case analysis	186	17.8	30.1	−12.3 (−21.7 to −4.1)	0.59 (0.40–0.86)

Values are standardized estimates from weighted two-part models; 95% CIs were derived from 1,000 patient-level bootstrap samples. Qualifying-episode continuation comprised AF time belonging to the uninterrupted eligibility-defining episode beyond day 56; the subsequent-episode analysis excluded this time but retained all eligible patients. Delayed outcome-window and alternative blanking-period analyses used cumulative AF days within their respective outcome periods.

At day 57, the qualifying episode remained ongoing in an estimated 4.1% under the prompt strategy and 13.6% under the deferred strategy, contributing 0.9 vs. 5.8 AF days and approximately 39% of the overall difference. After excluding this time, burden from distinct subsequent episodes remained lower under the prompt strategy (17.0 vs. 24.7 days; difference, −7.7; 95% CI, −14.9 to −1.1; mean ratio, 0.69; 95% CI, 0.47–0.97). Differences persisted when follow-up began on day 90 (−8.2 days; 95% CI, −15.4 to −1.7) or day 120 (−6.3 days; 95% CI, −12.2 to −0.8) (Table 3).

Among 168 patients with stable antiarrhythmic therapy, the difference was −12.5 days (95% CI, −22.2 to −3.6) (Table 3). Censoring and reweighting at the first post-strategy drug modification, cardioversion, or repeat ablation gave a difference of −10.4 days (95% CI, −18.9 to −2.5). The mean-ratio E-value was 2.78 (1.67 for the confidence limit); quantitative bias analysis indicated that substantial unmeasured imbalance would be required to explain the point estimate.

## Discussion

In this target-trial emulation, prompt conditional cardioversion for sustained early AF recurrence after PFA was associated with lower subsequent AF burden than 7-day deferral. The association extended beyond earlier termination of the qualifying episode: distinct later episodes, sustained recurrence, repeat cardioversion, and AF-related hospitalization also favored prompt management. Results were consistent across sensitivity analyses, and the exploratory landmark analysis suggested attenuation with delay. Repeat-ablation and safety estimates were imprecise. Because treatment was not randomized and empirical support for the deferred strategy was weaker, these findings support a potentially time-sensitive benefit of rhythm restoration but do not establish a definitive cardioversion window.

The principal PFA trials established effectiveness and procedural safety but evaluated outcomes after index ablation rather than management after sustained early recurrence (1, 2). Randomized comparisons showed PFA to be comparable with conventional thermal and cryoballoon ablation for paroxysmal AF (3, 5). Real-world EU-PORIA data also support durable PFA-based pulmonary-vein isolation (4). Our study addresses the later decision point at which sustained AF has already recurred. PFA-SHAM added a blinded sham-controlled comparison to the evidence base (6). That trial addressed benefit attributable to ablation, whereas our study examines postprocedural management among patients with sustained recurrence. The questions are complementary because rhythm-control decisions after an effective index procedure may still influence subsequent AF exposure.

Early recurrence after PFA is not uniformly benign. PFA-specific work has questioned a single undifferentiated blanking interval (7). An EHRA survey documented wide variation in how clinicians define and manage such recurrence (8). A secondary PULSED AF analysis further linked the timing and pattern of early recurrence to later outcomes (9). We add a comparison of operational cardioversion strategies rather than prognosis alone. The lack of detectable effect modification by recurrence timing suggests that the association was not confined to a narrow part of the 8-week window. This differs from timing-dependent prognostic reports, possibly because we studied a management effect rather than recurrence as a risk marker and restricted eligibility to postablation days 3–49. Continuous monitoring is central to interpretation. ADVANTAGE AF demonstrated burden-sensitive assessment with insertable monitors after PFA for persistent AF (10). Increasing first-line use of PFA will enlarge the population requiring standardized management of early recurrence (11). Burden distinguishes occasional short recurrences from prolonged exposure and is therefore more informative than a binary freedom-from-recurrence endpoint. Our study applies this outcome to a dynamic management question and found concordant continuous and threshold-based results.

One mechanism is immediate: earlier cardioversion shortens an episode that might otherwise enter the outcome window. The association with distinct later episodes also raises the possibility that prolonged AF promotes remodeling and further AF, consistent with evidence that early rhythm control can slow progression (12). This interpretation is clinically relevant because AF burden and episode duration are associated with healthcare use and quality of life (13). Cardioversion may interrupt a self-reinforcing period, but we did not measure remodeling or separate an attempted procedure from durable sinus-rhythm restoration. The landmark attenuation provides temporal coherence, although later landmarks contained fewer, selected patients who remained in AF and should be interpreted descriptively.

Transient biology remains plausible. PFA produces myocardial injury and inflammatory and coagulation responses despite its nonthermal mechanism (14). Some early episodes may resolve as acute effects subside. However, the association persisted after excluding qualifying-episode continuation and delaying outcome assessment, making a purely transient explanation less likely. Medication changes were also unlikely to explain the entire association. POWDER-AF2 found no benefit from continuing previously ineffective antiarrhythmic therapy after optimized isolation in selected persistent AF (15), and our estimates were consistent under stable-therapy and intervention-censoring analyses. This study adds continuous rhythm ascertainment, explicit conditional strategies, clone-censor-weight methods, episode decomposition, and landmark analyses to the literature on cardioversion timing after PFA.

For patient care, the results support early reassessment rather than passive acceptance of sustained AF during the blanking period. In a stable, adequately anticoagulated patient with continuously documented AF lasting at least 24 h, preparing for cardioversion within 48 h if AF persists may reduce later burden and acute-care use. Because spontaneous conversion remained adherent, this approach is conditional and does not require shocking every stable patient immediately. The findings do not apply to brief episodes, justify cardioversion without anticoagulation assessment, or prove that 48 h is optimal.

Burden-sensitive follow-up may be more informative than a recurrence label because AF duration is related to healthcare use and quality of life (13). Postablation pathways should also address equity because women are often underrepresented in ablation research, although comparative PFA data in women are reassuring (16). Institutions could standardize rhythm verification, symptom and rate assessment, anticoagulation, rescue criteria, and access to elective cardioversion. Such protocols should preserve individualized decisions and are not a guideline mandate.

Uncommon adverse outcomes cannot establish safety equivalence. PFA has encouraging bronchial safety data (17). Coronary spasm and hemolysis-associated acute kidney injury have nevertheless been reported (18, 19). The cohort was too small to compare rare events, and cardioversion should continue to follow established anticoagulation, sedation, and hemodynamic-safety standards.

This study was a single-center retrospective cohort with a modest sample size and selected patients with high-coverage continuous monitoring and sustained recurrence. Generalizability to shorter episodes, intermittent monitoring, more diverse populations, or different PFA workflows is uncertain. Target-trial emulation cannot reproduce randomization and depends on measured confounding, correct models, and positivity. Unmeasured symptom dynamics, clinician preference, atrial substrate, or care access may remain despite good measured balance and reassuring sensitivity analyses. Greater censoring and a lower effective sample size under deferral particularly limit that strategy's precision.

Monitoring platforms were heterogeneous, so nonwear and algorithmic differences may have caused measurement error despite common thresholds, adjudication, and observation weighting. Eligibility was anchored to AF lasting at least 24 h on postablation days 3–49; sensitivity analyses were supportive, but other episode patterns remain untested. Later drug changes, cardioversion, and repeat ablation were components of the total strategy effect; supportive analyses cannot isolate the biological effect of cardioversion. No rescue event occurred, so findings apply only to stable patients. Rare safety events and several secondary outcomes were underpowered. Landmark and effect-modification analyses were exploratory, and multiple related outcomes increase the possibility of chance findings.

A multicenter pragmatic randomized trial should compare prompt conditional cardioversion with protocolized short deferral in stable patients with sustained early post-PFA AF. Blinded adjudication and sham-informed patient-reported assessment would strengthen inference (6). Continuous monitoring should separate total burden, qualifying-episode continuation, and new episodes (10). Longer-term PFA outcome studies provide an important durability benchmark for such follow-up (20). Stratification by AF type, recurrence timing, PFA platform, atrial substrate, and antiarrhythmic therapy, with prespecified safety, quality-of-life, hospitalization, and cost outcomes, could identify who benefits and whether the relevant window is measured in hours or days.

Among continuously monitored patients with sustained early AF recurrence after first-time PFA, prompt conditional cardioversion was associated with lower subsequent AF burden and less recurrent rhythm-control use than 7-day deferral. The association extended beyond shortening the qualifying episode. These findings support standardized early reassessment and randomized validation but do not establish a mandatory cardioversion threshold.

## Data Availability

The original contributions presented in the study are included in the article/Supplementary Material, further inquiries can be directed to the corresponding author/s.
