# Supplementary material for: Prompt versus deferred conditional cardioversion strategies after early AF recurrence following pulsed field ablation
Source: Front Cardiovasc Med. 2026 Oct 28;13:1948373. doi: 10.3389/fcvm.2026.1948373 (PMC13751417; doi:10.3389/fcvm.2026.1948373)
Supplement: Supplementary file 1 [file Table1.docx]

**Supplementary Table 1. Baseline, procedural, time-zero, and rhythm-monitoring characteristics.**

| **Characteristic** | **Overall cohort (N=214)** | **Prompt strategy, weighted** | **Deferred strategy, weighted** | **ASMD** |
| --- | --- | --- | --- | --- |
| **Baseline characteristics** |  |  |  |  |
| **Age, years** | **64.3 ± 9.6** | **64.2 ± 9.5** | **64.5 ± 9.6** | **0.031** |
| Women | 91 (42.5) | 0.421 | 0.428 | 0.014 |
| BMI, kg/m² | 25.9 ± 3.2 | 25.9 ± 3.2 | 26.0 ± 3.2 | 0.031 |
| Persistent AF | 99 (46.3) | 0.46 | 0.465 | 0.01 |
| AF duration, years | 3.9 (1.8–7.2) | 3.8 (1.8–7.1) | 4.0 (1.9–7.3) | 0.038 |
| Preablation AF burden, % | 28.6 (14.2–51.8) | 28.2 (14.0–51.1) | 28.9 (14.4–52.2) | 0.027 |
| Previous cardioversion | 63 (29.4) | 0.297 | 0.292 | 0.011 |
| Hypertension | 129 (60.3) | 0.601 | 0.606 | 0.01 |
| Diabetes mellitus | 37 (17.3) | 0.176 | 0.172 | 0.011 |
| Coronary artery disease | 29 (13.6) | 0.138 | 0.134 | 0.012 |
| Chronic heart failure | 38 (17.8) | 0.175 | 0.18 | 0.014 |
| Previous stroke or TIA | 18 (8.4) | 0.086 | 0.083 | 0.011 |
| Chronic kidney disease | 16 (7.5) | 0.074 | 0.077 | 0.011 |
| Obstructive sleep apnea | 25 (11.7) | 0.119 | 0.115 | 0.012 |
| Left atrial volume index, mL/m² | 43.2 ± 10.1 | 43.1 ± 10.0 | 43.4 ± 10.1 | 0.03 |
| Left ventricular ejection fraction, % | 59.1 ± 7.8 | 59.2 ± 7.7 | 59.0 ± 7.8 | 0.026 |
| Active antiarrhythmic-drug therapy at index PFA | 149 (69.6) | 0.698 | 0.694 | 0.009 |
| Rate-control medication use | 128 (59.8) | 0.595 | 0.601 | 0.012 |
| Procedural characteristics |  |  |  |  |
| FARAPULSE system | 132 (61.7) | 0.69 | 0.695 | 0.011 |
| PulseSelect system | 82 (38.3) | 0.31 | 0.305 | 0.011 |
| Intraprocedural cardioversion | 94 (43.9) | 0.33 | 0.334 | 0.009 |
| Additional posterior-wall ablation | 43 (20.1) | 0.209 | 0.203 | 0.015 |
| Time-zero characteristics |  |  |  |  |
| Postablation day of recurrence | 18 (10–31) | 18 (10–31) | 18 (10–32) | 0.023 |
| Ventricular rate, beats/min | 112 ± 24 | 111.6 ± 23.8 | 112.4 ± 24.1 | 0.033 |
| EHRA class III or IV symptoms | 111 (51.9) | 0.515 | 0.522 | 0.014 |
| Symptomatic qualifying AF episode (EHRA II–IV) | 156 (72.9) | 0.726 | 0.733 | 0.016 |
| Emergency-department or inpatient care | 77 (36.0) | 0.356 | 0.363 | 0.015 |
| Acute heart-failure worsening | 22 (10.3) | 0.101 | 0.105 | 0.013 |
| Verified therapeutic anticoagulation | 201 (93.9) | 0.941 | 0.937 | 0.017 |
| Active antiarrhythmic-drug therapy at qualifying recurrence (time zero) | 162 (75.7) | 0.759 | 0.754 | 0.013 |
| Rate-control therapy at time zero | 139 (65.0) | 0.648 | 0.653 | 0.011 |
| Preablation AF-burden ascertainment (90-day window) |  |  |  |  |
| Prespecified monitoring window, days | 90 | 90 | 90 | 0 |
| Analyzable monitoring coverage, % | 95.8 (91.9-98.7) | 95.9 (92.0-98.8) | 95.7 (91.7-98.6) | 0.015 |
| Coverage >=90% | 181 (84.6) | 0.848 | 0.843 | 0.014 |
| Coverage 80% to <90% | 33 (15.4) | 0.152 | 0.157 | 0.014 |
| Longest uninterrupted monitoring gap, days | 2.0 (0.9-3.9) | 1.9 (0.9-3.8) | 2.0 (1.0-4.0) | 0.018 |
| Same predominant modality in preablation and outcome periods | 208 (97.2) | 0.973 | 0.971 | 0.011 |
| Common minimum AF episode duration counted, min | 2 | 2 | 2 | 0 |
| Postablation rhythm-monitoring characteristics (days 57-365) |  |  |  |  |
| Any implantable monitoring | 152 (71.0) | 0.714 | 0.708 | 0.014 |
| Implantable cardiac monitor | 121 (56.5) | 0.568 | 0.562 | 0.012 |
| Pacemaker | 19 (8.9) | 0.087 | 0.09 | 0.011 |
| Implantable cardioverter-defibrillator | 7 (3.3) | 0.034 | 0.032 | 0.011 |
| Cardiac resynchronization device | 5 (2.3) | 0.025 | 0.024 | 0.006 |
| Validated continuously worn ECG system | 62 (29.0) | 0.286 | 0.292 | 0.014 |
| Analyzable monitoring coverage, % | 96.2 (92.4-98.9) | 96.3 (92.6-99.0) | 96.1 (92.2-98.8) | 0.018 |
| Coverage >=90% | 186 (86.9) | 0.872 | 0.866 | 0.017 |
| Coverage 80% to <90% | 28 (13.1) | 0.128 | 0.134 | 0.017 |
| Longest uninterrupted monitoring gap, days | 1.8 (0.8-3.6) | 1.7 (0.8-3.5) | 1.8 (0.8-3.7) | 0.021 |
| Monitoring system changed during outcome period | 6 (2.8) | 0.029 | 0.027 | 0.012 |
| Common minimum AF episode duration counted, min | 2 | 2 | 2 | 0 |
| Rhythm-ascertainment quality control |  |  |  |  |
| Eligibility-defining episodes double-adjudicated | 214/214 (100) | NA | NA | NA |
| Preablation algorithm detections screened | 2,084 (100) | NA | NA | NA |
| Preablation detections independently double-read | 417/2,084 (20.0) | NA | NA | NA |
| Preablation false-positive detections excluded | 55/2,084 (2.6) | NA | NA | NA |
| Preablation interobserver agreement, % | 96 | NA | NA | NA |
| Preablation Cohen kappa (95% CI) | 0.91 (0.88-0.94) | NA | NA | NA |
| Outcome-period algorithm detections screened | 6,842 (100) | NA | NA | NA |
| Outcome-period detections independently double-read | 1,368/6,842 (20.0) | NA | NA | NA |
| Outcome-period false-positive detections excluded | 178/6,842 (2.6) | NA | NA | NA |
| Outcome-period interobserver agreement, % | 96.2 | NA | NA | NA |
| Outcome-period Cohen kappa (95% CI) | 0.92 (0.90-0.94) | NA | NA | NA |

Data are mean +/- SD, median (IQR), or n (%). Strategy-specific columns present standardized distributions after inverse-probability weighting. Monitoring modality was defined by the system contributing the greatest number of analyzable minutes in the corresponding period. ASMD indicates absolute standardized mean difference; NA, not applicable.

Missing values occurred for BMI in 2 patients (0.9%), AF duration in 3 (1.4%), left atrial volume index in 6 (2.8%), left ventricular ejection fraction in 3 (1.4%), serum potassium in 9 (4.2%), magnesium in 14 (6.5%), and creatinine in 7 (3.3%). Twenty-eight patients (13.1%) had at least one missing covariate.

Preablation AF burden was calculated during the 90 days immediately preceding the index PFA as total adjudicated AF minutes divided by total analyzable monitoring minutes. Postablation monitoring coverage was calculated during days 57-365 using the same formula and common minimum AF episode duration of 2 minutes. All patients had at least 80% analyzable coverage in both periods. Signal loss, device interruption, and wearable nonwear were excluded from the denominator; AF time was not imputed during monitoring gaps. All algorithm detections were screened by trained reviewers, and two electrophysiologists blinded to treatment strategy independently adjudicated all eligibility-defining episodes and a 20% random sample of detections, with disagreements resolved by consensus.

AF, atrial fibrillation; ASMD, absolute standardized mean difference; BMI, body mass index; CI, confidence interval; ECG, electrocardiogram; EHRA, European Heart Rhythm Association; IQR, interquartile range; NA, not applicable; PFA, pulsed-field ablation; TIA, transient ischemic attack.
