# Supplementary material for: Prompt versus deferred conditional cardioversion strategies after early AF recurrence following pulsed field ablation
Source: Front Cardiovasc Med. 2026 Oct 28;13:1948373. doi: 10.3389/fcvm.2026.1948373 (PMC13751417; doi:10.3389/fcvm.2026.1948373)
Supplement: Supplementary file 2 [file Table2.docx]

Supplementary Table 2. Procedural characteristics of the index pulsed-field ablation.

| **Characteristic** | **Overall (N=214)** | **FARAPULSE (n=132)** | **PulseSelect (n=82)** |
| --- | --- | --- | --- |
| Persistent AF | 99 (46.3) | 70 (53.0) | 29 (35.4) |
| General anesthesia | 153 (71.5) | 93 (70.5) | 60 (73.2) |
| Deep sedation | 61 (28.5) | 39 (29.5) | 22 (26.8) |
| Electroanatomic mapping used | 177 (82.7) | 112 (84.8) | 65 (79.3) |
| PVI performed | 214 (100) | 132 (100) | 82 (100) |
| Isolation after planned application set | 184 (86.0) | 115 (87.1) | 69 (84.1) |
| Additional PFA applications for residual PV conduction | 30 (14.0) | 17 (12.9) | 13 (15.9) |
| Final acute isolation of all PVs | 214 (100) | 132 (100) | 82 (100) |
| Waiting period, min | 20 [20–25] | 20 [20–25] | 20 [20–25] |
| Additional posterior-wall ablation | 43 (20.1) | 35 (26.5) | 8 (9.8) |
| Posterior-wall ablation among persistent AF patients | 43/99 (43.4) | 35/70 (50.0) | 8/29 (27.6) |
| Acute posterior-wall isolation achieved | 43/43 (100) | 35/35 (100) | 8/8 (100) |
| **Indications among posterior-wall ablation patients*** |  |  |  |
| AF duration ≥6 months | 31/43 (72.1) | 25/35 (71.4) | 6/8 (75.0) |
| Left atrial volume index ≥40 mL/m² | 27/43 (62.8) | 22/35 (62.9) | 5/8 (62.5) |
| Posterior-wall low-voltage substrate | 15/43 (34.9) | 13/35 (37.1) | 2/8 (25.0) |
| **Other adjunctive lesion sets*** |  |  |  |
| Cavotricuspid-isthmus ablation | 14 (6.5) | 9 (6.8) | 5 (6.1) |
| Mitral-isthmus ablation | 3 (1.4) | 2 (1.5) | 1 (1.2) |
| Superior vena cava isolation | 2 (0.9) | 1 (0.8) | 1 (1.2) |
| Focal atrial-tachycardia ablation | 2 (0.9) | 1 (0.8) | 1 (1.2) |
| Pulmonary-vein isolation alone | 161 (75.2) | 92 (69.7) | 69 (84.1) |
| Intraprocedural cardioversion | 94 (43.9) | 65 (49.2) | 29 (35.4) |
| Total procedure duration, min | 86 [70–106] | 82 [68–99] | 93 [76–113] |
| Left atrial dwell time, min | 55 [44–70] | 52 [42–66] | 61 [48–77] |
| Fluoroscopy time, min | 13.1 [8.4–19.5] | 11.9 [7.7–17.8] | 15.4 [9.8–22.1] |

Data are n (%) or median [IQR]. *Indications for posterior-wall ablation and other adjunctive lesion sets were not mutually exclusive. Posterior-wall ablation was performed only in patients with persistent AF. Application counts across platforms should not be directly compared because the platforms use different catheter configurations and energy-delivery sequences. AF indicates atrial fibrillation; PFA, pulsed-field ablation; PV, pulmonary vein; and PVI, pulmonary-vein isolation.
