# Supplementary material for: Prompt versus deferred conditional cardioversion strategies after early AF recurrence following pulsed field ablation
Source: Front Cardiovasc Med. 2026 Oct 28;13:1948373. doi: 10.3389/fcvm.2026.1948373 (PMC13751417; doi:10.3389/fcvm.2026.1948373)
Supplement: Supplementary file 3 [file Table3.docx]

Supplementary Table 3. Clinical characteristics of cardioversion for the qualifying AF recurrence.

| **Characteristic** | **Overall** | **Within 48 hours** | **>48 hours to day 7** | **After day 7** |
| --- | --- | --- | --- | --- |
| Patients undergoing cardioversion | 154 | 109 | 18 | 27 |
| Immediate sinus-rhythm restoration | 149/154 (96.8) | 106/109 (97.2) | 17/18 (94.4) | 26/27 (96.3) |
| Early AF reinitiation within 10 minutes* | 8/149 (5.4) | 5/106 (4.7) | 1/17 (5.9) | 2/26 (7.7) |
| Sinus rhythm at end of 30-minute observation | 145/154 (94.2) | 103/109 (94.5) | 17/18 (94.4) | 25/27 (92.6) |
| Direct oral anticoagulant | 140/154 (90.9) | 99/109 (90.8) | 17/18 (94.4) | 24/27 (88.9) |
| Vitamin K antagonist | 14/154 (9.1) | 10/109 (9.2) | 1/18 (5.6) | 3/27 (11.1) |
| Prior 3-week therapeutic anticoagulation verified | 145/154 (94.2) | 103/109 (94.5) | 17/18 (94.4) | 25/27 (92.6) |
| TEE before cardioversion | 14/154 (9.1) | 8/109 (7.3) | 2/18 (11.1) | 4/27 (14.8) |
| Thrombus detected on TEE | 0/14 (0) | 0/8 (0) | 0/2 (0) | 0/4 (0) |
| Anticoagulation continued for ≥4 weeks | 154/154 (100) | 109/109 (100) | 18/18 (100) | 27/27 (100) |
| Rescue cardioversion during days 0–7 | 0/214 (0) | 0 | 0 | NA |

Data are n/N (%). Cardioversion timing was measured from time zero. Immediate restoration was defined as sinus rhythm sustained for at least 30 seconds after a synchronized shock. *The denominator for early AF reinitiation was patients with immediate sinus-rhythm restoration. Acute procedural success was defined as sinus rhythm at the end of the 30-minute observation period. TEE was performed because uninterrupted therapeutic anticoagulation could not be verified in 9 patients and because of additional clinical concern for thrombus in 5 patients. AF indicates atrial fibrillation; NA, not applicable; and TEE, transesophageal echocardiography.
