# Supplementary material for: Prompt versus deferred conditional cardioversion strategies after early AF recurrence following pulsed field ablation
Source: Front Cardiovasc Med. 2026 Oct 28;13:1948373. doi: 10.3389/fcvm.2026.1948373 (PMC13751417; doi:10.3389/fcvm.2026.1948373)
Supplement: Supplementary file 4 [file Table4.docx]

Supplementary Table 4. Artificial censoring, weighting diagnostics, and covariate balance.

| **Diagnostic** | **Prompt strategy** | **Deferred strategy** | **Overall or comparison** |
| --- | --- | --- | --- |
| Clones assigned at time zero | 214 | 214 | 428 |
| Artificially censored | 76 (35.5%) | 127 (59.3%) | 203 (47.4%) |
| Between-strategy difference in artificial censoring | Reference | +23.8 percentage points | — |
| Uncensored through strategy window | 138 (64.5%) | 87 (40.7%) | 225 (52.6%) |
| Probability of strategy compatibility, median (IQR) | 0.65 (0.49–0.79) | 0.42 (0.27–0.60) | — |
| Probability range | 0.07–0.94 | 0.06–0.91 | 0.06–0.94 |
| Stabilized weight, median (IQR) | — | — | 1.03 (0.86–1.26) |
| Truncated weight range | — | — | 0.52–3.84 |
| Effective sample size | 124.7 | 72.9 | 197.6 |
| Effective sample size as percentage of assigned clones | 58.3% | 34.1% | 46.2% |
| Effective sample size as percentage of uncensored clones | 90.4% | 83.8% | 87.8% |
| Median post-weighting ASMD | — | — | 0.021 |
| Maximum ASMD before weighting | — | — | 0.418 |
| Covariates with ASMD ≥0.10 before weighting | — | — | 13 |
| Maximum ASMD after weighting | 0.054 | 0.057 | 0.057 |
| Covariates with ASMD ≥0.10 after weighting | 0 | 0 | 0 |

Data are n (%) unless otherwise indicated. Strategy-compatibility probabilities were estimated using separate strategy-specific pooled logistic models in 24-hour intervals. Denominator models included prespecified baseline and lagged time-varying covariates; continuous variables were modeled using restricted cubic splines with three knots, categorical variables as indicator terms, and time using interval indicators. Treatment-adherence and monitoring weights were multiplied and truncated at the 1st and 99th percentiles. Effective sample size was calculated as (Σw)²/Σw². The censoring difference is deferred minus prompt. ASMD indicates absolute standardized mean difference; IQR, interquartile range.
