# Supplementary material for: Prompt versus deferred conditional cardioversion strategies after early AF recurrence following pulsed field ablation
Source: Front Cardiovasc Med. 2026 Oct 28;13:1948373. doi: 10.3389/fcvm.2026.1948373 (PMC13751417; doi:10.3389/fcvm.2026.1948373)
Supplement: Supplementary file 5 [file Table5.docx]

Supplementary Table 5. Post-recurrence rhythm-control interventions through day 365.

| **Intervention** | **Overall, n (%)** | **Prompt strategy, % (95% CI)** | **Deferred strategy, % (95% CI)** | **Risk difference, percentage points (95% CI)** | **Risk ratio (95% CI)** |
| --- | --- | --- | --- | --- | --- |
| Any antiarrhythmic drug modification | 46 (21.5) | 19.3 (14.0–25.5) | 26.7 (19.8–34.5) | −7.4 (−16.0 to 1.4) | 0.72 (0.50–1.03) |
| Initiation | 12 (5.6) | 5.2 (2.6–8.8) | 7.5 (4.0–12.0) | −2.3 (−6.8 to 2.0) | 0.69 (0.33–1.35) |
| Discontinuation | 14 (6.5) | 6.8 (3.8–10.8) | 6.1 (3.0–10.4) | 0.7 (−4.0 to 5.4) | 1.11 (0.58–2.13) |
| Agent or drug-class switch | 8 (3.7) | 3.3 (1.3–6.1) | 5.5 (2.7–9.5) | −2.2 (−6.3 to 1.6) | 0.60 (0.26–1.28) |
| Dose adjustment ≥25% | 18 (8.4) | 7.6 (4.4–11.8) | 11.2 (6.8–16.6) | −3.6 (−9.3 to 1.8) | 0.68 (0.39–1.14) |
| Subsequent electrical cardioversion | 43 (20.1) | 22/138 (15.9)^*^ 15.8 (10.8–21.8) | 22/87 (25.3)^*^ 24.9 (18.1–32.5) | −9.1 (−18.1 to −0.6) | 0.63 (0.42–0.94) |
| Repeat ablation | 23 (10.7) | 12/138 (8.7)^*^ 8.7 (5.0–13.6) | 11/87 (12.6)^*^ 13.1 (8.2–19.1) | −4.4 (−11.3 to 2.0) | 0.66 (0.38–1.12) |

Antiarrhythmic drug modifications were assessed from time zero through day 365. Component categories were not mutually exclusive. Subsequent cardioversion and repeat ablation were counted after day 7 to provide a common post-strategy starting point. *For these two procedural outcomes, the first line in each strategy column gives the unweighted n/N (%) among clones remaining compatible with the assigned strategy through day 7; the second line gives the standardized risk (95% CI). Because patients were cloned to both strategies at baseline, strategy-specific clone counts are not mutually exclusive. Other strategy-specific values are standardized estimates. All 95% CIs were obtained from 1,000 patient-level bootstrap samples.
